# Real-world efficacy and safety of disitamab vedotin monotherapy or in combination with PD-1 inhibitors in locally advanced or metastatic upper tract urothelial carcinoma: a multicenter retrospective study

**DOI:** 10.3389/fimmu.2025.1699538

**Published:** 2026-01-06

**Authors:** Shuaipeng He, Cheng Wang, Xuan Li, Can Li, Dan Li, Biao Zhang, Jianghou Wan, Zhongjin Yue, Gongjin Wu, Su Zhang, Junhai Ma, Panfeng Shang

**Affiliations:** 1 Department of Urology, The Second Hospital of Lanzhou University, Lanzhou, China; 2 Department of Urology, The First Hospital of Lanzhou University, Lanzhou, China

**Keywords:** antibody–drug conjugate, disitamab vedotin, HER2, immunotherapy, real-world study, upper tract urothelial carcinoma

## Abstract

**Objective:**

Disitamab vedotin (RC48) is a HER2-targeted antibody–drug conjugate (ADC) that can induce immunogenic cell death and enhance antitumor immunity. Preclinical data suggest synergistic activity with PD-1 inhibitors, but its benefit in upper tract urothelial carcinoma (UTUC) remains unclear. This study evaluated the efficacy and safety of RC48, as monotherapy or combined with PD-1 blockade, in advanced UTUC.

**Methods:**

We conducted a multicenter retrospective study of patients with locally advanced or metastatic UTUC treated with RC48 ± PD-1 inhibitors. Baseline features and adverse events (AEs) were summarized descriptively. Objective response rate (ORR) and disease control rate (DCR) were calculated with exact 95% confidence intervals. Progression-free survival (PFS) and overall survival (OS) were estimated by Kaplan–Meier and compared with the log-rank test. Multivariable Cox models included covariates significant in univariable analyses.

**Results:**

A total of 41 patients were analyzed (9 monotherapy, 32 combination). Median follow-up was 16.5 months. The 12-month OS rate was 92.3%, and the 24-month OS rate was 63.2%; median OS was not reached. Median PFS was 12.6 months, with 6- and 12-month PFS rates of 78.0% and 56.4%, respectively. In an exploratory subgroup analysis, the 12-month PFS rate was 68.9% for first-line treatment versus 36.5% for later lines (p = 0.031). Among 22 patients with measurable lesions, ORR was 40.9% (9/22) and DCR was 81.8% (18/22). In the overall cohort of 41 patients, grade ≥3 AEs occurred in 18.8% (6/32) of patients in the combination group, including one grade 4 Stevens–Johnson syndrome.

**Conclusion:**

RC48, used alone or in combination with PD-1 inhibitors, showed preliminary antitumor activity with manageable toxicity in patients with locally advanced or metastatic UTUC in this multicenter real-world cohort.

## 1 Introduction

Upper tract urothelial carcinoma (UTUC) is a distinct subtype of urothelial carcinoma (UC), representing approximately 5%–10% of all UC cases. The management of UTUC remains particularly challenging, especially when the tumor progresses to a muscle-invasive stage. About 30% of patients with initially localized UTUC eventually develop metastatic disease, and 12%–16% present with distant metastases at the time of diagnosis (1, 2). Although radical nephroureterectomy (RNU) has long been regarded as the standard first-line treatment for muscle-invasive UTUC, multiple real-world studies and systematic reviews have shown that patients remain at substantial risk of recurrence and metastasis after surgery. Reported rates of intravesical recurrence (IVR) range from 22% to 47%, while recurrence at regional or distant sites occurs in approximately 30% of cases (2–4).

For elderly patients who are unfit for surgery, as well as those with locally advanced or metastatic disease, traditional platinum-based chemotherapy—such as the gemcitabine plus cisplatin (GC) regimen—remains the mainstay of treatment, yielding a median progression-free survival (PFS) of 7.7 months and a median overall survival (mOS) of 14.0 months. However, these regimens are frequently accompanied by substantial systemic toxicities, which limit tolerability in certain patients and ultimately restrict their overall therapeutic efficacy (5, 6).

In recent years, immune checkpoint inhibitors (ICIs) and antibody–drug conjugates (ADCs) have demonstrated promising efficacy in the treatment of advanced UC. Inhibitors targeting the PD-1/PD-L1 axis—such as pembrolizumab and atezolizumab—have been integrated into standard therapeutic regimens for UC, providing durable clinical responses and meaningful survival benefits in a subset of patients (7). Concurrently, ADCs targeting membrane proteins such as HER2 achieve selective cytotoxicity by delivering potent cytotoxic payloads into tumor cells with high target expression. These agents have demonstrated favorable efficacy and manageable safety profiles across multiple UC subgroups (8).

However, most clinical evidence to date has focused on bladder cancer, whereas data specific to UTUC remain limited. HER2 expression in UTUC is generally lower and more heterogeneous than in bladder cancer, which complicates the predictive value of HER2-targeted ADCs in this setting (9, 10). As a result, precision therapeutic strategies targeting HER2 in UTUC are still under exploration.

RC48 (Disitamab Vedotin) is a novel antibody-drug conjugate (ADC) that specifically targets the HER2 receptor and delivers the cytotoxic payload monomethyl auristatin E (MMAE) into tumor cells, thereby exerting potent antitumor activity. Recent studies have demonstrated that MMAE-based payloads not only exert direct cytotoxic effects but also have the capacity to induce immunogenic cell death (ICD). ICD is characterized by hallmark signals such as calreticulin (CRT) externalization, extracellular ATP release, and the liberation of high-mobility group box 1 (HMGB1), which collectively recruit and activate dendritic cells (DCs), enhance antigen presentation, and ultimately trigger tumor-specific immune responses (11, 12).

Moreover, ADCs may further potentiate adaptive antitumor immunity by inducing primary tumor cell death, releasing damage-associated molecular patterns (DAMPs), activating T cells, and modulating the tumor immune microenvironment. These mechanisms provide a strong biological rationale for combining ADCs with immune checkpoint inhibitors (ICIs). In the phase Ib/II RC48-C014 study, which enrolled 41 patients, disitamab vedotin combined with toripalimab achieved an objective response rate (ORR) of 73.2%, with a median progression-free survival (PFS) of 9.3 months and a median overall survival (OS) of 33.1 months, indicating a potential synergistic effect between RC48 and immune checkpoint inhibition (13).

Preliminary efficacy has also been observed in patients with HER2-negative or low-expressing disease (IHC 0 or 1+). In a multicenter retrospective study involving 27 UC patients with HER2-negative or low HER2 expression—including 20 cases of UTUC—partial responses (PR) were achieved in 8 patients (30.8%), while 12 patients (46.2%) exhibited stable disease (SD), resulting in an objective response rate (ORR) of 30.8% and a disease control rate (DCR) of 76.9%. The median progression-free survival (PFS) and overall survival (OS) were 7.4 and 13.8 months, respectively, with 1-year PFS and OS rates of 29.1% and 57.2%. Most treatment-related adverse events were grade 1–2 and were generally well tolerated (14). Nevertheless, most pivotal clinical trials to date have focused primarily on bladder cancer populations, with UTUC patients markedly underrepresented in both ICI and ADC studies. Robust evidence to support the extrapolation of these therapeutic strategies to UTUC remains limited, and no prospective trials specifically evaluating RC48 in UTUC have been conducted thus far.

To address this evidence gap, the present study aimed to evaluate the efficacy and safety of RC48, administered either as monotherapy or in combination with an ICI, in patients with UTUC through a multicenter retrospective design. The study further examined the influence of HER2 expression status and treatment regimens on clinical outcomes, providing preliminary evidence to support the stratified application of RC48 in this population. These findings are anticipated to guide future prospective investigations and offer new therapeutic insights for patients with refractory UTUC, with important clinical and translational implications.

## 2 Methods

### 2.1 Study design

This multicenter retrospective clinical study included patients with pathologically confirmed UTUC who received treatment at the First Hospital and the Second Hospital of Lanzhou University. The study period extended from March 2023 to November 2024. The median follow-up duration was 16.5 months (range, 6.6–27.0 months). For each patient, follow-up began at the initiation of RC48 treatment and continued until death or the last follow-up on June 1, 2025, whichever occurred first.

### 2.2 Inclusion and exclusion criteria

The inclusion criteria were as follows: (1) age between 18 and 90 years at treatment initiation; (2) histologically confirmed diagnosis of primary UTUC; (3) stage T2–T4 disease, or disease with regional lymph node or distant metastases; (4) receipt of at least two cycles of RC48 treatment; (5) prior platinum-based chemotherapy (e.g., gemcitabine plus cisplatin, GC regimen) or treatment-naïve status; (6) availability of complete baseline clinical and imaging data, with at least two CT or MRI evaluations; and (7) at least one valid follow-up record with available data for progression-free survival (PFS) or overall survival (OS).

The exclusion criteria were as follows: (1) receipt of fewer than two cycles of RC48 treatment; (2) missing HER2 expression data; (3) presence of concurrent malignancies or unknown primary tumors; and (4) loss to follow-up precluding efficacy or survival evaluation.

### 2.3 Treatment regimens

Disitamab vedotin (RC48) was administered at 2.0 mg/kg intravenously every 2 weeks (Q2W). The combination regimen was “RC48 plus a PD-1 inhibitor (tislelizumab or toripalimab)”. When combined with PD-1 inhibitors, toripalimab 240 mg or tislelizumab 200 mg was given intravenously every 3 weeks (Q3W) according to institutional practice and product labeling. Dose reductions, delays, and treatment discontinuations were allowed at the discretion of the treating physicians and were recorded together with reasons for modification. RC48 was administered either as first-line or second-line treatment. Patients who received RC48 as second-line therapy had previously experienced disease progression or intolerance after first-line platinum-based chemotherapy, typically the gemcitabine plus cisplatin (GC) regimen.

Clinical data were abstracted from electronic medical records and follow-up notes, including demographics, baseline disease characteristics, imaging assessments, HER2 immunohistochemistry status, detailed treatment exposure (start/stop dates, cycles, and modifications), clinical outcomes, and adverse events. Adverse events were graded according to CTCAE version 5.0.

### 2.4 HER2 assessment

HER2 expression was assessed by immunohistochemistry (IHC) and interpreted by experienced pathologists in accordance with the *2022 Clinical Pathological Expert Consensus on HER2 Testing in Chinese Urological Tumors*. Based on IHC scoring criteria, patients were stratified into two groups: HER2-negative/low expression (0/1+) and HER2-high expression (2+/3+). Fluorescence *in situ* hybridization (FISH) testing was not performed; all HER2 classifications were determined solely by IHC results (15).

### 2.5 Tumor response evaluation

Tumor response was assessed according to the Response Evaluation Criteria in Solid Tumors, version 1.1 (RECIST v1.1). The objective response rate (ORR) was defined as the proportion of patients achieving a complete response (CR) or partial response (PR), while the disease control rate (DCR) was defined as the proportion achieving CR, PR, or stable disease (SD). Response assessments were conducted only in the 22 patients with measurable lesions, including those with unresected or metastatic disease. Radiologic assessments were performed every 6–8 weeks (q6–8w) according to institutional practice. Imaging evaluations were interpreted by experienced radiologists and independently reviewed by two investigators. In cases of discrepancy, a third senior radiologist adjudicated the findings to reach consensus.

### 2.6 Endpoint definitions (ORR, DCR, PFS, OS)

The primary endpoint was progression-free survival (PFS), defined as the time from the initiation of RC48 treatment to the first radiologically confirmed disease progression (according to RECIST v1.1) or death from any cause, whichever occurred first. Patients without documented disease progression or death at the end of follow-up were censored at the date of their last follow-up.

Secondary endpoints included overall survival (OS), defined as the time from treatment initiation to death from any cause, with censoring at the last follow-up for patients who were alive. Additional secondary endpoints comprised the objective response rate (ORR), disease control rate (DCR), and adverse events (AEs). AEs were graded according to the Common Terminology Criteria for Adverse Events (CTCAE), version 5.0.

### 2.7 Statistical analysis

All statistical analyses were performed using R software (version 4.5.0). Baseline characteristics and adverse events were summarized as median (interquartile range, IQR) or mean (standard deviation, SD) for continuous variables, and as counts (percentages) for categorical variables. Between-group comparisons were conducted using Fisher’s exact or χ² tests for categorical variables and either the *t* test or Wilcoxon rank-sum test for continuous variables, as appropriate. The objective response rate (ORR) and disease control rate (DCR) were reported with exact binomial 95% confidence intervals (CIs); when zero counts occurred, Fisher’s exact test was applied, and absolute differences with corresponding 95% CIs were presented.

Progression-free survival (PFS) was defined from the first RC48 dose to radiographic progression or death from any cause; overall survival (OS) from the first dose to death from any cause. Patients without an event were censored at the last disease assessment or contact. Survival curves were estimated by the Kaplan–Meier method and compared with the log-rank test.

Multivariable Cox proportional hazards models were fitted to evaluate factors associated with progression-free survival (PFS). Covariates included in the multivariable model were those that achieved statistical significance in the univariable analyses, while ensuring an adequate events-per-variable ratio. The proportional hazards assumption was assessed using Schoenfeld residuals, both globally and for individual covariates, with visual inspection of the residual plots. Multicollinearity was examined using variance inflation factors (VIFs). Missing data, if present, were addressed through complete-case analysis, and sensitivity analyses were conducted when appropriate. A two-sided *p* value <0.05 was considered statistically significant. Model diagnostics, including Schoenfeld residual plots for each covariate and the global test, are provided in **Supplementary Figure S2**.

Analyses were locked on June 1, 2025.

## 3 Results

### 3.1 Baseline characteristics

A total of 41 eligible patients were included in the study, comprising 35 from the Second Hospital of Lanzhou University and 6 from the First Hospital of Lanzhou University. Detailed baseline characteristics are summarized in **Table 1**. The median age was 64 years (range, 47–87), and 25 patients (61.0%) were male while 16 (39.0%) were female.

**Table 1:** Characteristics of the patients (n = 41).

Characteristics	Values				
	All patients	RC48 monotherapy(n=9)	RC48 + PD-1(n=32)	HER2 2+/3+(n=21)	HER2 0/1+ (n=20)
Age (years)					
Median (range)	64.0 (47–87)	63.0 (53-73)	65.5 (47-87)	65.0 (47-87)	63.5 (52-81)
Sex, n (%)					
Male	25 (61.0)	4 (44.4)	21 (65.6)	15 (71.4)	10 (50.0)
Female	16 (39.0)	5 (55.6)	11 (34.4)	6 (28.6)	10 (50.0)
Primary lesion, n (%)					
Renal pelvis	16 (39.0)	3 (33.3)	13 (40.6)	8 (38.1)	8 (40.0)
Ureter	23 (56.1)	5 (55.6)	18 (56.3)	11 (52.4)	12 (60.0)
Ureteropelvic junction	2 (4.9)	1 (11.1)	1 (3.1)	2 (9.5)	0
Metastasis site, n (%)					
Lymph node metastasis	16 (39.0)	2 (22.2)	14 (43.8)	7 (33.3)	9 (45.0)
Bone	6 (14.6)	2 (22.2)	4 (12.5)	3 (14.3)	3 (15.0)
Liver	5 (12.2)	2 (22.2)	3 (9.4)	3 (14.3)	2 (10.0)
Peritoneal metastasis	2 (4.9)	0	2 (6.3)	2 (9.5)	0
HER2 IHC, n (%)					
0/1+	20 (48.8)	3 (33.3)	17 (53.1)	0	20 (100.0)
2+/3+	21 (51.2)	6 (66.7)	15 (46.9)	21 (100.0)	0
Treatment, n (%)					
RC48 monotherapy	9 (22.0)	9 (100.0)	0	6 (28.6)	3 (15.0)
RC48 +PD-1	32 (78.0)	0	32 (100.0)	15 (71.4)	17 (85.0)
Prior therapy, n (%)					
Prior platinum-based chemotherapy	16 (39.0)	5 (55.6)	11 (34.4)	6 (28.6)	10 (50.0)
Prior locoregional curative treatments, n (%)	2 (4.9)	0	2 (6.3)	0	2 (10.0)
Radical nephroureterectomy, n (%)	30 (73.2)	7 (77.8)	23 (71.9)	14 (66.7)	16 (80.0)

Data are presented as median (range) or number (%). Percentages are calculated using the group total as the denominator. RC48 + PD-1, disitamab vedotin combined with PD-1 inhibitor(tislelizumab, n = 18; toripalimab, n = 14). IHC, immunohistochemistry.

The primary tumor site was located in the ureter in 23 patients (56.1%), the renal pelvis in 16 (39.0%), and the ureteropelvic junction in 2 (4.9%). Eleven patients (26.8%) presented with visceral metastases, and 16 (39.0%) had regional lymph node involvement.

Immunohistochemistry (IHC) results showed that 21 patients (51.2%) had high HER2 expression (IHC 2+ or 3+), whereas 20 (48.8%) exhibited low (IHC 1+) or negative (IHC 0) expression.

Regarding treatment regimens, 9 patients (22.0%) received RC48 monotherapy and 32 (78.0%) received RC48 in combination with a PD-1 inhibitor (toripalimab or tislelizumab). RC48 was administered as first-line therapy in 25 patients (61.0%) and as second-line therapy following platinum-based chemotherapy (e.g., gemcitabine plus cisplatin) in 16 (39.0%). In addition, 11 patients (26.8%) had not undergone radical surgery before receiving RC48 treatment.

Overall, the baseline characteristics demonstrated clinical heterogeneity, with a balanced distribution of HER2 expression and a predominance of combination immunotherapy, reflecting real-world treatment patterns for advanced UTUC.

### 3.2 Efficacy

**Figure 1:**
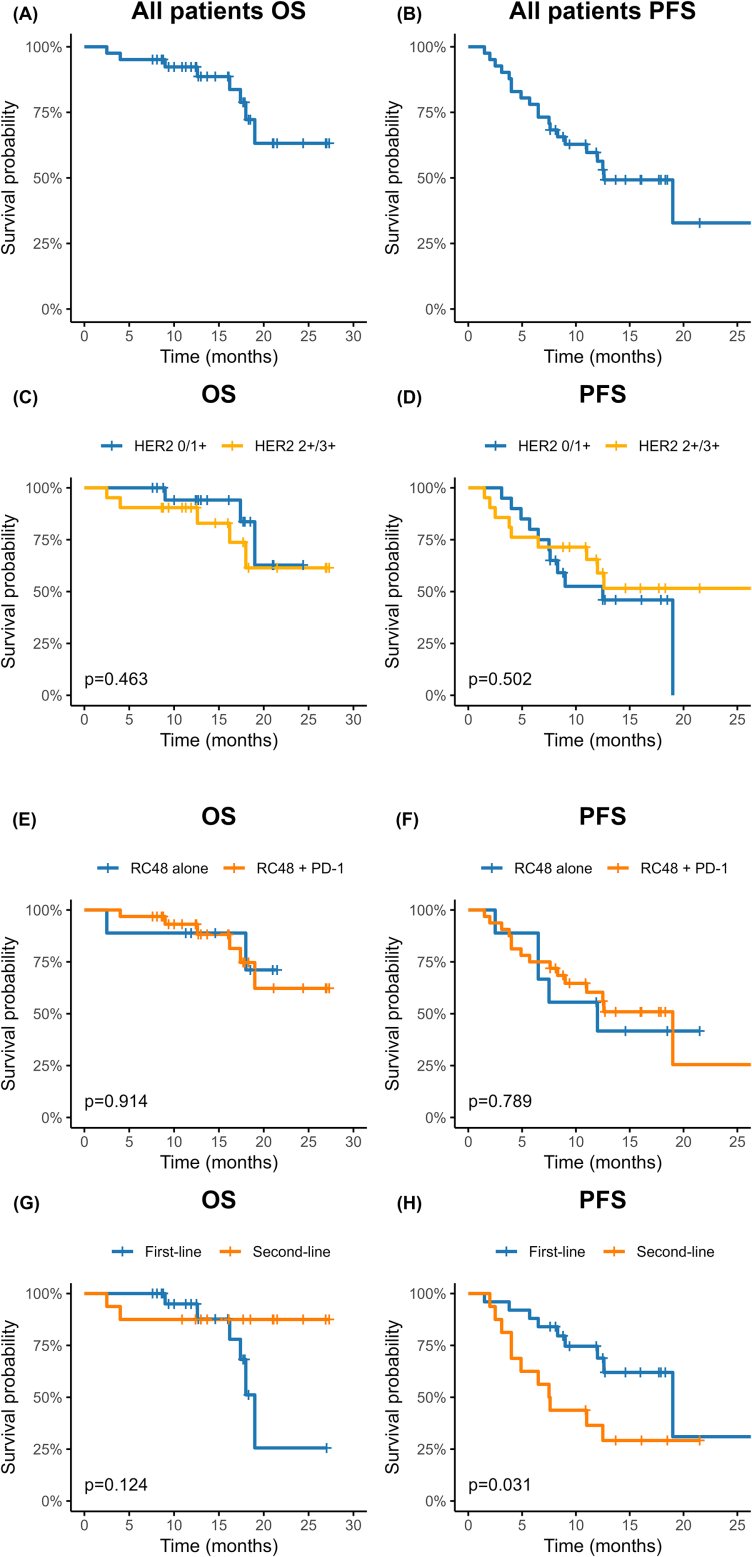
Kaplan–Meier survival analysis of 41 patients with locally advanced or metastatic upper tract urothelial carcinoma (UTUC) treated with RC48 with or without PD-1 inhibitors. **(A)** Overall survival (OS) of all patients. **(B)** Progression-free survival (PFS) of all patients. **(C)** OS stratified by HER2 status. **(D)** PFS stratified by HER2 status. **(E)** OS stratified by treatment regimen (RC48 monotherapy vs. RC48 plus PD-1). **(F)** PFS stratified by treatment regimen. **(G)** OS stratified by treatment line (first-line vs. second-line). **(H)** PFS stratified by treatment line.

Kaplan–Meier survival analysis was performed for 41 patients with locally advanced or metastatic UTUC, with a median follow-up duration of 16.5 months (range, 6.6–27.0 months) (**Figure 1**). The 12-month overall survival (OS) rate for the entire cohort was 92.3% (95% CI, 84.3–100), and the 24-month OS rate was 63.2% (95% CI, 43.5–91.7). The median OS was not reached (95% CI, 19.0–not reached). For PFS, the median duration was 12.6 months (95% CI, 9.0–not reached), with 6-month and 12-month PFS rates of 78.0% (95% CI, 66.4–91.8) and 56.4% (95% CI, 42.5–74.7), respectively. Despite the limited sample size, the 12-month PFS rate remained relatively favorable at 56.4%.

Subgroup analysis revealed that the 12-month progression-free survival (PFS) rate was significantly higher in the first-line treatment group compared with the second-line group (68.9%, 95% CI, 51.8–91.6 vs. 36.5%, 95% CI, 18.8–70.6; log-rank *p* = 0.031). The 12-month PFS rate in the RC48 plus PD-1 group was 60.3% (95% CI, 44.9–81.0), compared with 41.7% (95% CI, 18.5–94.0) in the monotherapy group, although the difference was not statistically significant (*p* = 0.789). Similarly, the 12-month PFS rate did not differ between patients with low/negative HER2 expression (IHC 0/1+) and those with high expression (IHC 2+/3+) (52.5%, 95% CI, 33.9–81.4 vs. 58.9%, 95% CI, 40.3–86.2; *p* = 0.502).

**Figure 2:**
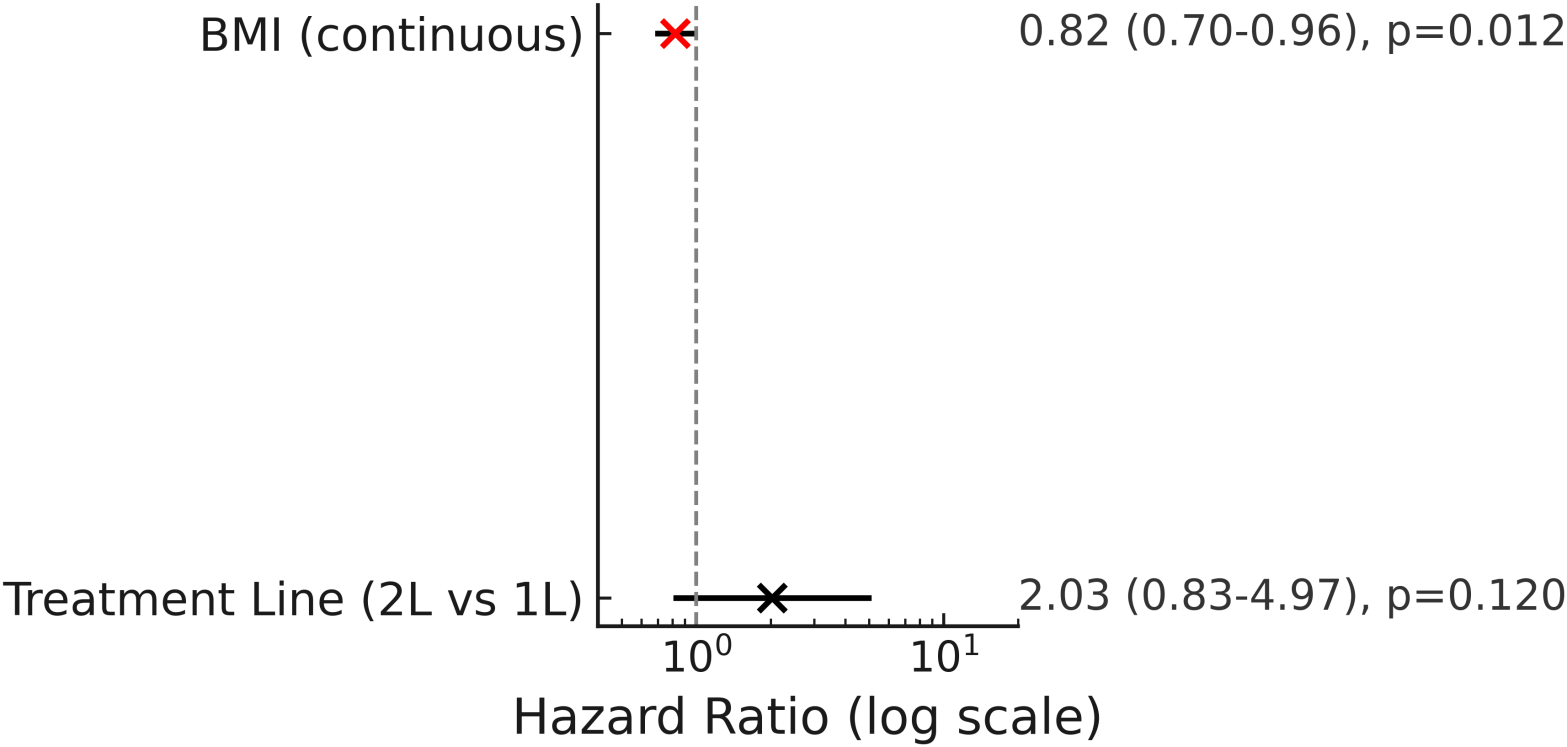
Forest plot of multivariable Cox regression analysis of progression-free survival (PFS). Hazard ratios (HRs) with 95% confidence intervals (CIs) are shown on a logarithmic scale; the vertical dashed line indicates HR = 1.

Univariable Cox regression analysis identified body mass index (BMI) (HR = 0.81, 95% CI, 0.70–0.94; *p* = 0.005) and treatment line (HR = 2.57, 95% CI, 1.06–6.21; *p* = 0.036) as factors significantly associated with progression-free survival (PFS). Higher BMI was correlated with longer PFS, whereas patients receiving second-line therapy exhibited a higher risk of disease progression compared with those receiving first-line treatment. Other variables, including HER2 status, T stage, and lymph node metastasis, were not statistically significant. In the multivariable Cox regression model, BMI remained an independent protective factor (HR = 0.82, 95% CI, 0.70–0.96; *p* = 0.012), while second-line treatment continued to show a trend toward increased risk (HR = 2.03, 95% CI, 0.83–4.97; *p* = 0.120), although this did not reach statistical significance (**Table 2**; **Figure 2**). The univariable forest plot is provided in the **Supplementary Materials** (**Supplementary Figure S1**).

**Table 2:** Univariable and multivariable Cox regression analysis of progression-free survival (PFS).

Variable	HR	CI_lower	CI_upper	P_value	Model
BMI	0.82	0.70	0.96	0.012*	Multivariable
BMI	0.81	0.70	0.94	0.005**	Univariable
HER2_status (HER2 2+/3+)	0.74	0.31	1.79	0.505	Univariable
Lymph_node_metastasis (Yes)	0.91	0.36	2.33	0.850	Univariable
T_stage_group (T3–T4)	1.43	0.57	3.62	0.445	Univariable
age_group (≥65)	0.5	0.20	1.27	0.147	Univariable
diabetes (Yes)	1.42	0.41	4.93	0.584	Univariable
hypertension (Yes)	0.83	0.33	2.09	0.696	Univariable
treatment_line (Second-line vs First-line)	2.03	0.83	4.97	0.120	Multivariable
treatment_line (Second-line vs First-line)	2.57	1.06	6.21	0.036*	Univariable
treatment_regimen (RC48 + PD-1 vs RC48 alone)	0.87	0.31	2.39	0.782	Univariable
tumor_grade (High vs Low)	0.93	0.30	2.75	0.863	Univariable
visceral_metastasis (Yes)	2.15	0.83	5.57	0.116	Univariable

Hazard ratios (HRs) and 95% confidence intervals (CIs) were calculated for each variable. Both univariable and multivariable Cox regression models were applied to identify potential prognostic factors. Significant associations were indicated by *p* < 0.05 (*), *p* < 0.01 (**).

Model diagnostics revealed no evidence of multicollinearity and no violations of the proportional hazards assumption. Schoenfeld residual plots were consistent with these findings (**Supplementary Figure S2**).

**Figure 3:**
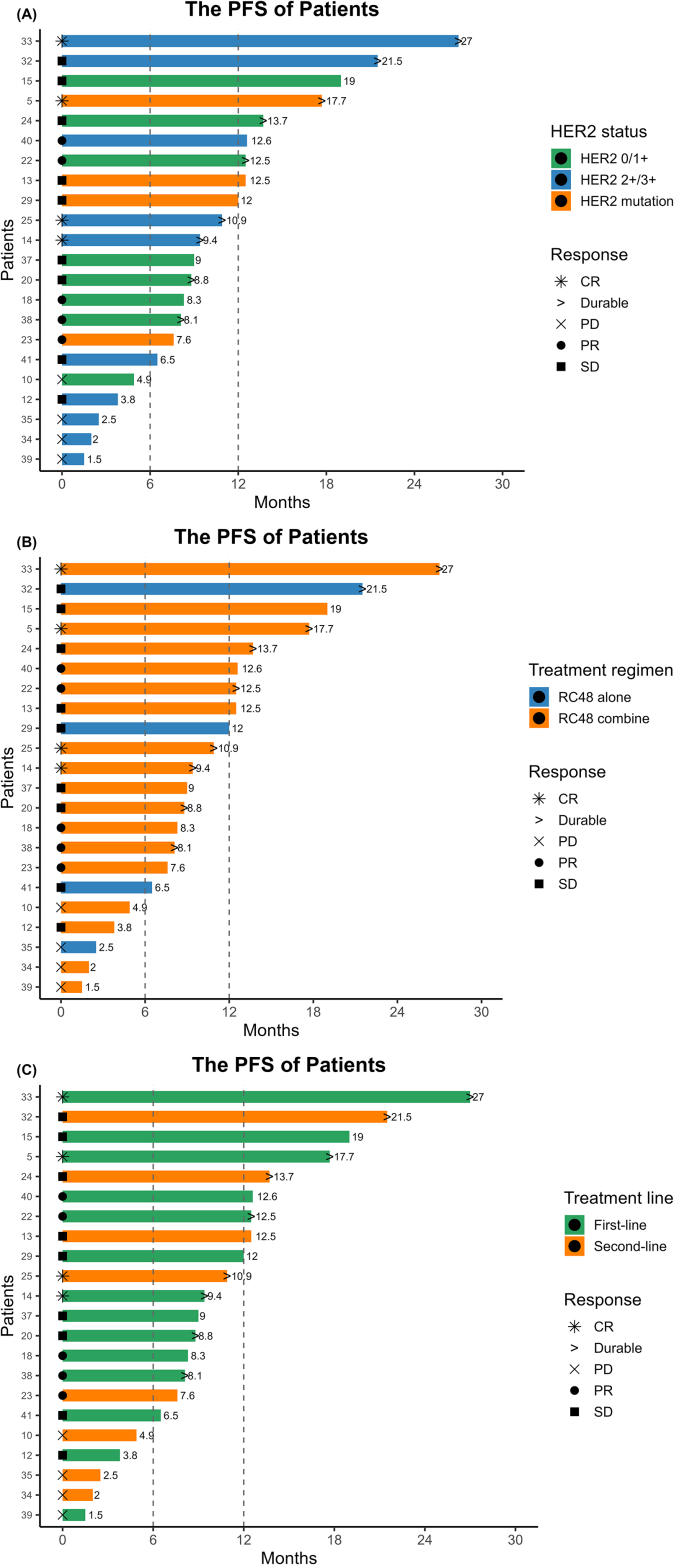
**(A)** stratified by HER2 status (HER2 0/1+, HER2 2+/3+, and HER2 mutation); **(B)** stratified by treatment regimen (RC48 alone vs RC48 combined); **(C)** stratified by treatment line (first-line vs second-line). Symbols denote best response [complete response (CR), partial response (PR), stable disease (SD), progressive disease (PD)]; numbers indicate exact months; arrows represent patients with ongoing treatment at the time of analysis. Vertical dashed lines mark the 6- and 12-month landmarks.

**Figure 4:**
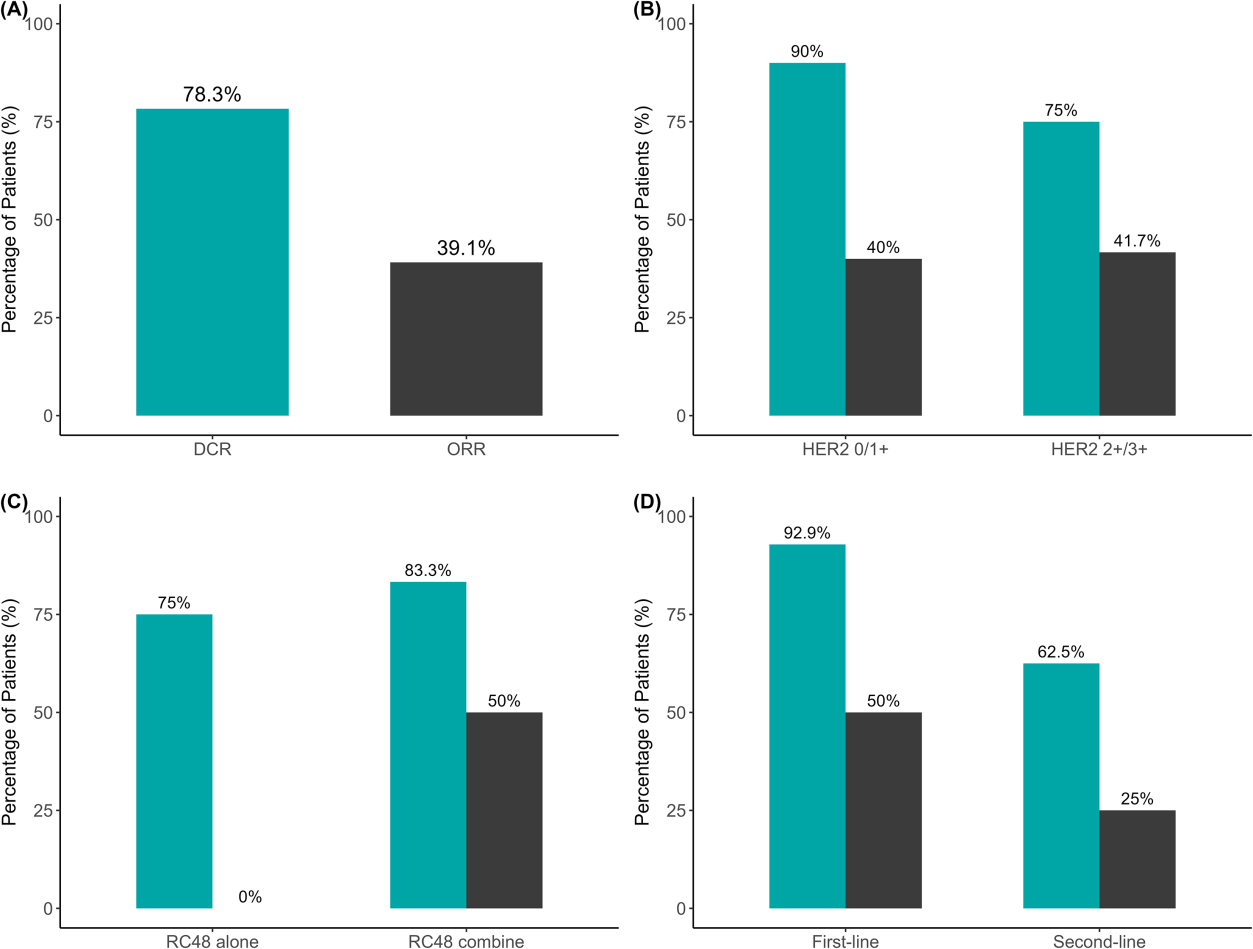
Objective response rate (ORR) and disease control rate (DCR) in evaluable patients and subgroups. **(A)** Overall ORR and DCR in evaluable patients. **(B)** ORR and DCR stratified by HER2 expression (0/1+ vs. 2+/3+). **(C)** ORR and DCR stratified by treatment regimen (RC48 monotherapy vs. RC48 plus PD-1). **(D)** ORR and DCR stratified by treatment line (first-line vs. second-line). Percentages are displayed above each bar. ORR was defined as the proportion of patients achieving CR or PR, while DCR included patients with CR, PR, or SD. N values are provided in the Results section.

Among the 22 patients evaluable for tumor response, the overall objective response rate (ORR) was 40.9%, and the disease control rate (DCR) was 81.8% (**Figures 3**, **4**).

In subgroup analyses by treatment line, patients receiving first-line therapy achieved a higher ORR than those treated in the second-line setting (50.0% [7/14] vs. 25.0% [2/8]), although the difference was not statistically significant (*p* = 0.380; OR = 0.35, 95% CI, 0.03–2.94). The DCR was also higher in the first-line group (92.9% vs. 62.5%), with a *p* value approaching statistical significance (*p* = 0.117; OR = 0.14, 95% CI, 0.002–2.25).

Among the 22 patients with measurable disease, the ORR was 50.0% (9/18) in the RC48 plus PD-1 group and 0.0% (0/4) in the RC48 monotherapy group. The absolute difference was 50 percentage points (Newcombe 95% CI, −20.0% to 71.0%; Fisher’s exact *p* = 0.115). Given the small denominator in the monotherapy subgroup, this comparison was underpowered and should be interpreted as hypothesis-generating. The DCR was comparable between the two groups (83.3% [15/18] vs. 75.0% [3/4]; *p* = 1.000; OR = 1.62, 95% CI, 0.02–31.44).

HER2 subgroup analysis revealed comparable objective response rates (ORRs) between patients with low/negative HER2 expression (IHC 0/1+) and those with high expression (IHC 2+/3+) (40.0% [4/10] vs. 41.7% [5/12]; *p* = 1.000; OR = 1.07, 95% CI, 0.14–8.24). The disease control rate (DCR) was numerically higher in the low/negative expression group compared with the high-expression group (90.0% vs. 75.0%), although the difference was not statistically significant (*p* = 0.594; OR = 0.35, 95% CI, 0.006–5.35).

Despite the limited sample size, these findings are consistent with the possibility that earlier application of RC48 may be associated with improved outcomes, but they should be interpreted cautiously and regarded as hypothesis-generating. Notably, in this small cohort HER2 expression level did not appear to markedly influence treatment efficacy; patients with low/negative HER2 expression even showed a slightly higher DCR than those with high expression. However, given the small subgroup sizes and the reliance on immunohistochemistry alone for HER2 assessment, these observations are descriptive only and cannot establish that RC48 benefit is independent of HER2 status.

Of particular interest, six patients underwent repeated HER2 assessments during disease progression or recurrence, of whom five (83.3%) exhibited dynamic changes in HER2 status. For instance, one patient classified as HER2 1+ at surgery converted to HER2 3+ upon bladder recurrence and subsequently achieved a complete response (CR) after five cycles of RC48 plus toripalimab. Another patient with baseline HER2 1+ converted to HER2–0 following bladder relapse after combination therapy. A third patient maintained HER2 1+ at baseline and after surgery but showed an increase to HER2 2+ upon recurrence, achieving prolonged stable disease (SD) lasting 12.5 months under RC48 treatment. In the neoadjuvant setting, one patient shifted from HER2 1+ on biopsy to 2+ after nephrectomy. Another patient with concomitant bladder cancer and liver metastasis demonstrated HER2 2+ on initial biopsy and HER2 3+ after radical nephroureterectomy, maintaining stable disease for 6 months before progression and subsequent death. Details of HER2 dynamic conversion and corresponding treatment responses are provided in **Supplementary Table S1** as exploratory findings.

Comparisons between first-line and later-line therapy may be subject to immortal time bias; a landmark/time-dependent analysis will be considered in future work, and these results should be interpreted as hypothesis-generating.

### 3.3 Adverse events

Among all 41 patients who received RC48 treatment, 30 (73.2%) experienced adverse events (AEs), of whom 6 (14.6%) developed grade ≥3 AEs, all within the combination therapy group. In the RC48 monotherapy group (n = 9), 7 patients experienced mild to moderate AEs, mainly including nausea, alopecia, elevated transaminases, hyperglycemia, and anemia, with no grade ≥3 AEs observed (**Table 3**).

**Table 3:** Summary of treatment-related adverse events (AEs) in all patients and subgroups.

Adverse events	All patients (n = 41)		RC48 monotherapy (n = 9)		RC48 + PD-1 (n = 32)	
	All grades n (%)	Grade ≥3 n (%)	All grades n (%)	Grade ≥3 n (%)	All grades n (%)	Grade ≥3 n (%)
Any adverse event	30 (73.2)	6 (14.6)	7 (77.8)	0	23 (71.9)	6 (18.8)
Pruritus	2 (4.9)	0	0	0	2 (6.3)	0
Fatigue	1 (2.4)	0	0	0	1 (3.1)	0
Stevens–Johnson syndrome (SJS)	1 (2.4)	1 (2.4)	0	0	1 (3.1)	1 (3.1)
Vomiting	3 (7.3)	2 (4.8)	0	0	3 (9.4)	2 (6.3)
Peripheral neuropathy	8 (19.5)	0	1 (11.1)	0	7 (21.9)	0
Nausea	14 (34.1)	0	3 (33.3)	0	11 (34.4)	0
Alopecia	12 (29.3)	0	2 (22.2)	0	10 (31.3)	0
Hyperglycemia	10 (24.4)	3 (7.2)	2 (22.2)	0	8 (25.0)	3 (9.4)
Elevated transaminases	9 (22.0)	0	2 (22.2)	0	7 (21.9)	0
Anemia	4 (9.8)	0	2 (22.2)	0	2 (6.3)	0

Summary of treatment-related adverse events in all patients (n = 41), comparing RC48 monotherapy (n = 9) and RC48 combined with PD-1 inhibitors (n = 32). Percentages are calculated based on the total number of patients in each group (All patients, n = 41; RC48 monotherapy, n = 9; RC48 + PD-1, n = 32). AEs were graded according to CTCAE version 5.0. SJS: Stevens–Johnson syndrome.

In contrast, among the 32 patients treated with RC48 plus a PD-1 inhibitor, 23 developed AEs. The most common events were nausea, alopecia, elevated liver enzymes, hyperglycemia, and peripheral neuropathy. Six patients developed grade ≥3 AEs, including Stevens–Johnson syndrome (SJS), vomiting, and severe hyperglycemia. Three patients discontinued treatment due to toxicity.

Of the 3 patients with grade ≥3 hyperglycemia, one had a prior history of diabetes, with fasting glucose levels increasing markedly from 8.36 mmol/L to 14.08 mmol/L. The other two patients developed new-onset grade 3 hyperglycemia during therapy despite normal baseline glucose levels.

Importantly, one case of immune-related SJS was observed. This patient began RC48 plus toripalimab therapy on October 24, 2023, for postoperative retroperitoneal lymph node metastases. After two cycles, on January 9, 2024, the patient developed extensive mucocutaneous lesions and was diagnosed with grade 4 SJS according to NCI CTCAE criteria. Treatment with RC48 and PD-1 inhibitor was immediately discontinued, and systemic corticosteroids along with supportive care were initiated. The patient’s condition improved but no further antitumor therapy was administered, and the patient eventually died of disease progression in December 2024.

Overall, most AEs in the RC48 monotherapy group were grade ≤2 and well tolerated. In the combination therapy group, the incidence of severe toxicities was higher, with some leading to treatment discontinuation. Nevertheless, the adverse events were generally manageable, and the overall safety profile was acceptable.

## 4 Discussion

The results demonstrated encouraging preliminary outcomes, with 12-month progression-free survival (PFS) and overall survival (OS) rates of 56.4% and 92.3%, respectively. These findings suggest potential clinical activity in this real-world cohort; however, the interpretation of survival outcomes should remain cautious given the limited sample size and observational study design.

Subgroup analyses revealed no statistically significant differences in PFS or OS between patients with high HER2 expression (IHC 2+/3+) and those with low/negative expression (IHC 0/1+), nor between the RC48 monotherapy group and the RC48 plus PD-1 combination group; however, these comparisons are clearly underpowered and should be regarded as descriptive and hypothesis-generating rather than evidence of equivalence.

Among patients with measurable disease, combination therapy with RC48 plus a PD-1 inhibitor was associated with an objective response rate (ORR) of 50.0% (9/18), whereas no objective responses were observed in the small RC48 monotherapy subgroup (0/4). The absolute difference between groups was 50 percentage points (Newcombe 95% CI, −20.0% to 71.0%; Fisher’s exact p = 0.115). Given the very small sample size in the monotherapy subgroup and the fact that the study was not designed or powered for a formal comparison between regimens, this finding should be considered exploratory and hypothesis-generating rather than confirmatory. In an exploratory analysis, patients with HER2-negative or low expression showed a numerically higher disease control rate (DCR) than those with high expression (90.0% vs. 75.0%), but this difference was not statistically significant (p = 0.594; OR = 0.35, 95% CI, 0.006–5.35) and should be interpreted with particular caution given the small subgroup sizes and the reliance on immunohistochemistry alone for HER2 assessment.

Among patients who received RC48 as first-line therapy, more favorable outcomes were observed. The 12-month PFS rate appeared higher in the first-line group than in the second-line group (*p* = 0.031). Within the evaluable cohort, the objective response rate (ORR) was 50.0% (7/14) in the first-line group versus 25.0% (2/8) in the second-line group, although the difference did not reach statistical significance (*p* = 0.380; OR = 0.35, 95% CI, 0.03–2.94). The disease control rate (DCR) was also numerically higher in the first-line group (92.9% vs. 62.5%), although this difference did not reach statistical significance (*p* = 0.117; OR = 0.14, 95% CI, 0.002–2.25). Given the small sample size and imbalance between treatment lines, these findings should be interpreted as exploratory and hypothesis-generating rather than confirmatory.

Further univariable Cox regression analysis revealed a significant association between body mass index (BMI) and PFS (*p* = 0.005). Multivariable analysis further confirmed BMI as an independent protective factor (*p* = 0.012). In addition, patients who received RC48 as second-line therapy showed a trend toward an increased risk of disease progression, but this association did not reach statistical significance (p = 0.120) and should be interpreted cautiously, as the study was not designed or powered to formally compare treatment lines and larger cohorts will be required for validation.

From a clinical perspective, many patients with UTUC experience a decline in renal function following surgery, which limits their eligibility for conventional platinum-based first-line chemotherapy. In this context, RC48 has a relatively manageable toxicity profile and may offer a broader therapeutic window for patients with advanced or metastatic disease (16). Mechanistically, disitamab vedotin is a HER2-directed antibody–drug conjugate that delivers a microtubule-disrupting vedotin payload. Preclinical studies have shown that HER2-targeted ADCs of this class can exert cytotoxic effects beyond strongly HER2-overexpressing cells via a bystander effect, which offers a plausible biological explanation for their activity in tumors with heterogeneous HER2 expression. However, these mechanisms were not directly assessed in the present cohort, and, given that HER2 status was evaluated solely by immunohistochemistry without FISH or molecular profiling, our clinical observations across HER2 strata should be interpreted as descriptive rather than as evidence that treatment benefit is independent of HER2 expression (17, 18). In addition, vedotin-based ADCs have been reported to induce immunogenic cell death, enhance antigen presentation, and modulate antitumor immune responses, providing a rationale for combination with PD-1/PD-L1 blockade. While this framework is scientifically consistent with the use of disitamab vedotin plus PD-1 inhibitors, these immunologic effects were not measured in our study and should be regarded purely as contextual background rather than as mechanistic proof of the clinical outcomes observed in this small, retrospective real-world cohort (19, 20).

Wei et al. conducted a two-center real-world study evaluating disitamab vedotin combined with PD-1 inhibitors for locally advanced bladder UC, reporting an objective response rate (ORR) of 88.9% (8/9) with no grade 4–5 adverse events (21). In our cohort, the ORR for the RC48 plus PD-1 subgroup was 50.0% (7/14), suggesting antitumor activity of this regimen in UTUC; however, cross-disease comparisons should be interpreted with caution given the small sample size and the biological and anatomical differences between upper tract and bladder tumors. Taken together, these observations indicate that our study adds complementary real-world data within the broader urothelial cancer spectrum and further underscores the need for prospective studies specifically designed for UTUC.

Notably, among the small subset of patients who underwent repeated HER2 assessments, 83.3% exhibited changes in HER2 status. This observation is consistent with reports of substantial intratumoral and temporal heterogeneity in HER2 expression and may partly contribute to the difficulty of using a single baseline IHC result to stratify outcomes, although the numbers in our cohort are too limited to draw firm conclusions (22).

In this study, higher BMI was independently associated with a lower risk of disease progression in patients with UTUC. This observation is consistent with prior cohort studies from the CROES-UTUC international registry and a large single-center series, which reported improved oncologic outcomes in patients with overweight or obesity compared with those with normal BMI (23, 24). These findings support the concept of an “obesity paradox” in UTUC, whereby better nutritional and muscle reserves and a less catabolic systemic state may contribute to improved tolerance of treatment and slower disease progression. Future prospective studies incorporating detailed body composition assessments (e.g., CT-based or DXA-derived measures) are warranted to clarify the underlying mechanisms and to avoid unjustified therapeutic restrictions in patients with higher BMI.

In terms of safety, a pooled analysis of two phase II clinical trials (RC48-C005 and RC48-C009) reported that approximately 58 patients experienced grade ≥3 treatment-related adverse events (TRAEs), with an incidence of nearly 40%, primarily hematologic and gastrointestinal toxicities that were overall manageable. Consistent with these clinical trials, our study also demonstrated an acceptable safety profile, with grade ≥3 adverse events occurring in 18.8% of patients (6/32), all within the combination therapy group. This finding was comparable to the results reported by Wei et al., whose study focused on patients with locally advanced or metastatic bladder cancer, whereas our cohort included those with locally advanced or metastatic upper tract urothelial carcinoma (UTUC). In Wei et al.’s study, nine patients experienced manageable toxicities, including 22.2% (2/9) grade III–IV events and no grade IV–V adverse events (21, 25). Notably, hyperglycemia was observed in some patients, suggesting that blood glucose monitoring and additional management may be required, particularly in those with pre-existing diabetes. In addition, one patient developed Stevens–Johnson syndrome (SJS) after treatment with RC48 plus toripalimab. Although such severe dermatologic events appear to be rare, similar cases have been reported with toripalimab-based regimens, underscoring the importance of close monitoring for rash or mucosal involvement and prompt drug discontinuation with initiation of systemic corticosteroids when SJS is suspected (26).

This study suggested that RC48 may have antitumor activity in some UTUC patients with non-high HER2 expression, but these observations are exploratory and must be interpreted with caution. Conventional anti-HER2 strategies have primarily focused on patients with IHC 2+/3+ or FISH-positive tumors. In our cohort and in other real-world reports, responses were observed across different HER2 expression categories; however, given the small subgroup sizes and the fact that HER2 status in this study was assessed solely by immunohistochemistry without FISH or molecular profiling, these findings should be regarded as descriptive and cannot be taken as evidence that treatment benefit is independent of HER2 expression.

In summary, RC48, used alone or in combination with PD-1 inhibitors, showed preliminary antitumor activity and an acceptable safety profile in this multicenter real-world cohort of patients with locally advanced or metastatic UTUC. Responses were observed across different HER2 expression categories, but the limited sample size, retrospective design, and reliance on immunohistochemistry alone for HER2 assessment preclude firm conclusions regarding HER2-related treatment effects or any superiority of specific treatment settings. These exploratory findings should therefore be regarded as hypothesis-generating and support further prospective evaluation of RC48-based regimens in larger, well-characterized UTUC populations.

This study has several limitations. First, its retrospective design introduces inevitable selection and information biases, and the small sample size (41 patients, 22 with measurable lesions) greatly limits the power of subgroup analyses and the precision of ORR and DCR estimates. Second, the lack of a randomized control group or propensity score adjustment means that causal inferences about the efficacy of RC48 or the impact of treatment line and HER2 status cannot be drawn. Third, HER2 and molecular profiling were incomplete: assessment relied solely on immunohistochemistry without routine FISH or next-generation sequencing, and overall few OS events occurred during a median follow-up of 16.5 months, leaving survival endpoints immature and associated with wide confidence intervals. Future prospective studies with larger cohorts, comprehensive biomarker profiling, and longer follow-up, including patient-reported outcomes, are needed to better define the role of RC48-based regimens in UTUC.

## 5 Conclusion

RC48, used alone or in combination with PD-1 inhibitors, showed preliminary antitumor activity and an acceptable safety profile in this multicenter real-world cohort of patients with locally advanced or metastatic UTUC. Given the small sample size, imbalances between treatment regimens, and reliance on immunohistochemistry alone for HER2 assessment, all subgroup findings, including those by treatment line and HER2 expression, should be regarded as exploratory and hypothesis-generating rather than definitive. Larger prospective studies with standardized biomarker profiling are needed to confirm these observations and to clarify the optimal role of RC48-based regimens in the management of UTUC.

## Data Availability

The raw data supporting the conclusions of this article will be made available by the authors, without undue reservation.

## Supplementary material

Supplementary Figure 1 Forest plot of univariable Cox regression analysis of progression-free survival (PFS). Hazard ratios (HRs) with 95% confidence intervals (CIs) are shown on a logarithmic scale; the vertical dashed line indicates HR = 1.

Supplementary Figure 2 Scaled Schoenfeld residual plots for the multivariable Cox model. **(A)** BMI; **(B)** treatment line. Solid curves represent smoothed β(t) over time; dashed lines indicate 95% confidence bands.

Supplementary Table 1 Dynamic changes in HER2 expression during disease progression or recurrence and corresponding clinical responses (exploratory analysis).
