# Supplementary figures and images for: Real-world efficacy and safety of disitamab vedotin monotherapy or in combination with PD-1 inhibitors in locally advanced or metastatic upper tract urothelial carcinoma: a multicenter retrospective study
Source: Front Immunol. 2026 Jan 6;16:1699538. doi: 10.3389/fimmu.2025.1699538 (PMC12816211; doi:10.3389/fimmu.2025.1699538)

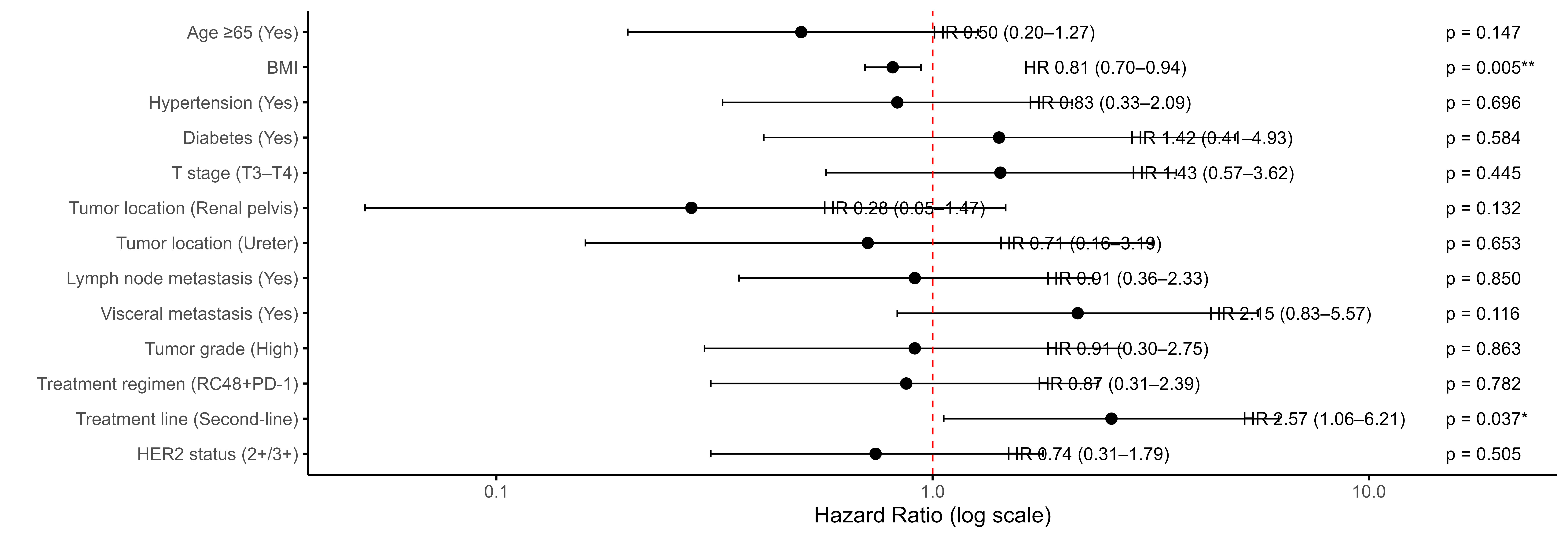

Supplement: Supplementary Figure 1 — Forest plot of univariable Cox regression analysis of progression-free survival (PFS). Hazard ratios (HRs) with 95% confidence intervals (CIs) are shown on a logarithmic scale; the vertical dashed line indicates HR = 1. [file Image1.tiff]

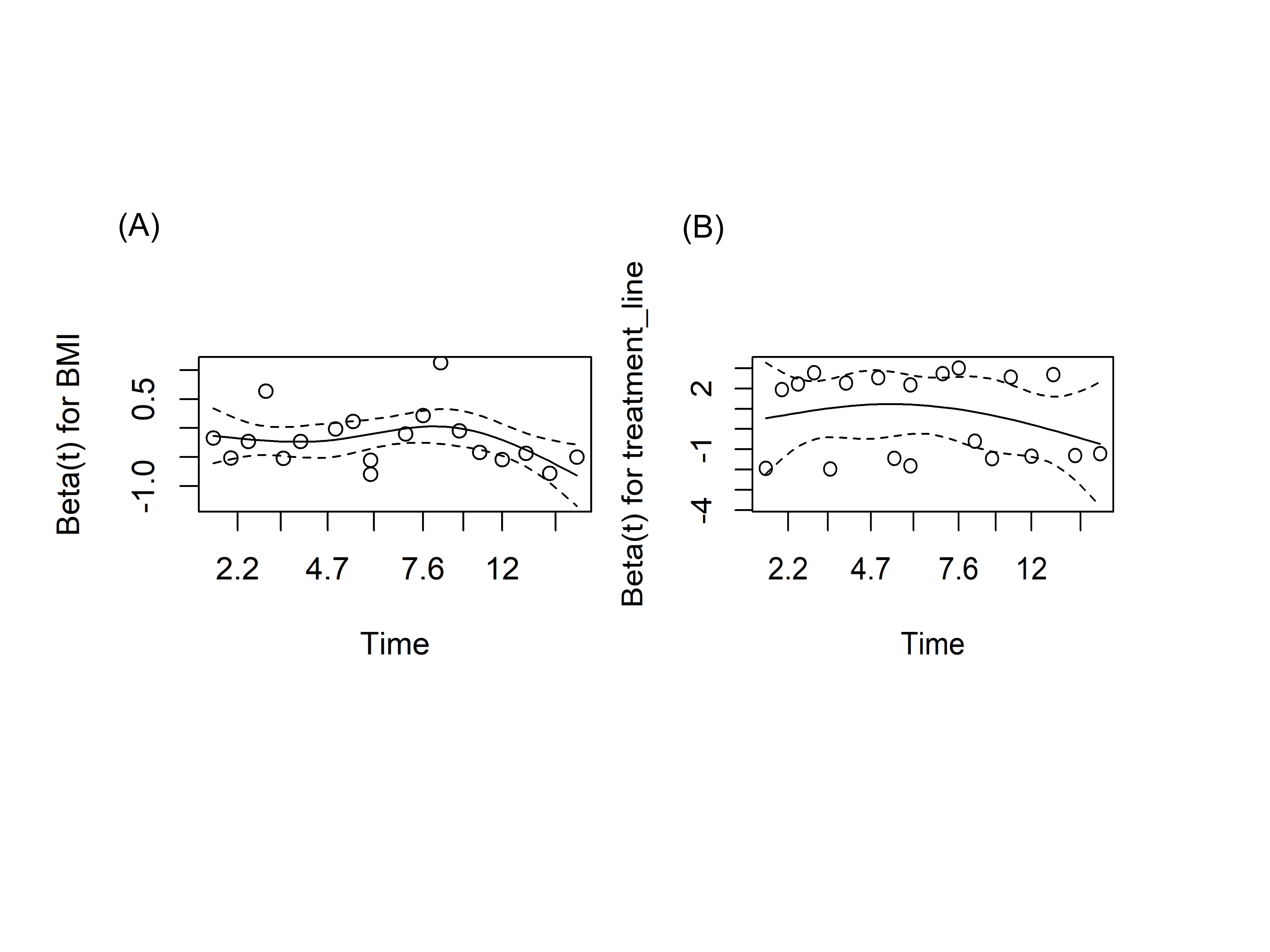

Supplement: Supplementary Figure 2 — Scaled Schoenfeld residual plots for the multivariable Cox model. (A) BMI; (B) treatment line. Solid curves represent smoothed β(t) over time; dashed lines indicate 95% confidence bands. [file Image2.jpeg]
