# Supplementary material for: Real-world efficacy and safety of disitamab vedotin monotherapy or in combination with PD-1 inhibitors in locally advanced or metastatic upper tract urothelial carcinoma: a multicenter retrospective study
Source: Front Immunol. 2026 Jan 6;16:1699538. doi: 10.3389/fimmu.2025.1699538 (PMC12816211; doi:10.3389/fimmu.2025.1699538)
Supplement: Supplementary Table 1 — Dynamic changes in HER2 expression during disease progression or recurrence and corresponding clinical responses (exploratory analysis). [file Table1.docx]

**Supplementary Table S1.**
Dynamic changes in HER2 expression during disease progression or recurrence and corresponding clinical responses (exploratory analysis).

| Case | Time Point 1 (Specimen) | HER2 IHC | Time Point 2 (Specimen) | HER2 IHC | Treatment Regimen | Best Response (RECIST v1.1) | PFS (months) |
| --- | --- | --- | --- | --- | --- | --- | --- |
| 1 | Surgery | 1+ | Bladder recurrence | 3+ | RC48 + Toripalimab | CR | 17.7 |
| 2 | Surgery | 1+ | Bladder relapse | 0 | RC48 + Toripalimab | Not evaluable | 3.1 |
| 3 | Surgery | 1+ | Post-surgery recurrence | 2+ | RC48 | SD | 12.5 |
| 4 | Biopsy (neoadjuvant) | 1+ | Post-nephrectomy | 2+ | RC48 + Toripalimab | PR | 7.6 |
| 5 | Biopsy | 2+ | Post-RNU (liver metastasis) | 3+ | RC48 + Toripalimab | SD | 12 |

**Supplementary Table S1.** HER2, human epidermal growth factor receptor 2; RC48, disitamab vedotin; Toripalimab, PD-1 inhibitor.
Best response was assessed per RECIST v1.1.
Patients with unavailable target lesions were labeled as “Not evaluable.”
Data are presented as descriptive and exploratory findings only, due to limited sample size and imbalance between subgroups.
